# Elucidating Conformation and Hydrogen-Bonding Motifs of Reactive Thiourea Intermediates

**DOI:** 10.1021/acscatal.2c03382

**Published:** 2022-10-05

**Authors:** Amelie A. Ehrhard, Lucas Gunkel, Sebastian Jäger, Arne C. Sell, Yuki Nagata, Johannes Hunger

**Affiliations:** Max-Planck Institute for Polymer Research, Ackermannweg 10, 55128 Mainz, Germany

**Keywords:** organo-catalysis, femtosecond IR spectroscopy, ab initio molecular dynamics simulations, density functional theory, hydrogen-bond dynamics

## Abstract

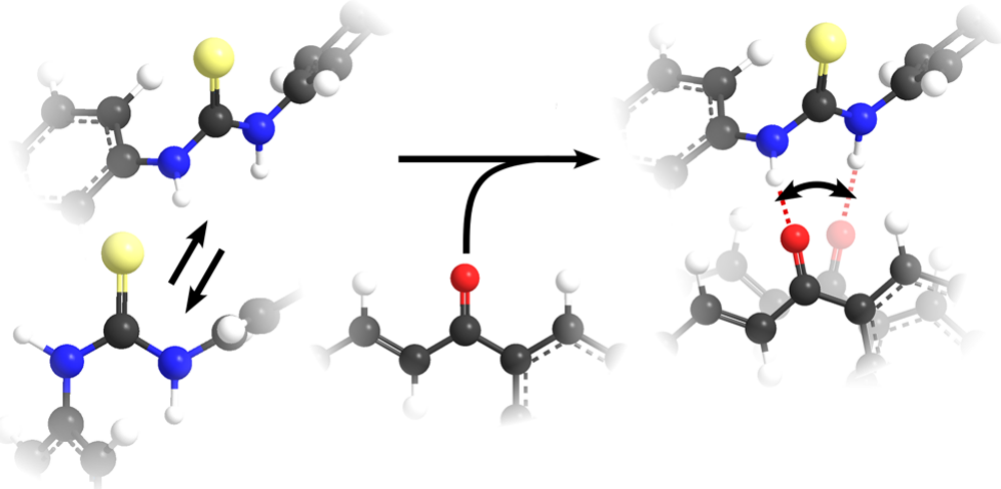

Substituted diphenylthioureas (DPTUs) are efficient hydrogen-bonding organo-catalysts, and substitution of DPTUs has been shown to greatly affect catalytic activity. Yet, both the conformation of DPTUs in solution and the conformation and hydrogen-bonded motifs within catalytically active intermediates, pertinent to their mode of activation, have remained elusive. By combining linear and ultrafast vibrational spectroscopy with spectroscopic simulations and calculations, we show that different conformational states of thioureas give rise to distinctively different N–H stretching bands in the infrared spectra. In the absence of hydrogen-bond-accepting substrates, we show that vibrational structure and dynamics are highly sensitive to the substitution of DPTUs with CF_3_ groups and to the interaction with the solvent environment, allowing for disentangling the different conformational states. In contrast to bare diphenylthiourea (0CF-DPTU), we find the catalytically superior CF_3_-substituted DPTU (4CF-DPTU) to favor the *trans*–*trans* conformation in solution, allowing for donating two hydrogen bonds to the reactive substrate. In the presence of a prototypical substrate, DPTUs in *trans*–*trans* conformation hydrogen bond to the substrate’s C=O group, as evidenced by a red-shift of the N–H vibration. Yet, our time-resolved infrared experiments indicate that only one N–H group forms a strong hydrogen bond to the carbonyl moiety, while thiourea’s second N–H group only weakly interacts with the substrate. Our data indicate that hydrogen-bond exchange between these N–H groups occurs on the timescale of a few picoseconds for 0CF-DPTU and is significantly accelerated upon CF_3_ substitution. Our results highlight the subtle interplay between conformational equilibria, bonding states, and bonding lifetimes in reactive intermediates in thiourea catalysis, which help rationalize their catalytic activity.

## Introduction

Thioureas have been shown to catalyze a wide range of organic transformations.^1−8^ In these catalytic conversions, hydrogen-bond formation of thiourea’s NH groups to electrophiles can efficiently activate the reacting electrophiles.^6,8,9^ The catalytic conversion rates in thiourea catalysis have been shown to be rather insensitive to the choice of the reactant, making thioureas widely applicable catalysts.^10^ Reaction rates can be markedly enhanced by substitution at the phenyl moiety:^10^ Trifluoromethyl substitution of diphenylthiourea^11^ greatly enhances reaction rates and yields.^10,12^ This enhancement has been primarily ascribed to electronic effects as the electron-withdrawing trifluoromethyl groups increase the acidity of the N–H groups,^9,13^ enhance π–π interactions,^4,14^ and enhance the polarity of the ortho-protons of the phenyl ring.^15^ Additionally, the choice of the solvent can affect catalytic efficiency.^16^ Yet, substitution at the phenyl ring (e.g., trifluoromethyl) and solvents can also affect conformational equilibria and thereby affect catalytic activity.^17−19^

**Figure 1:**
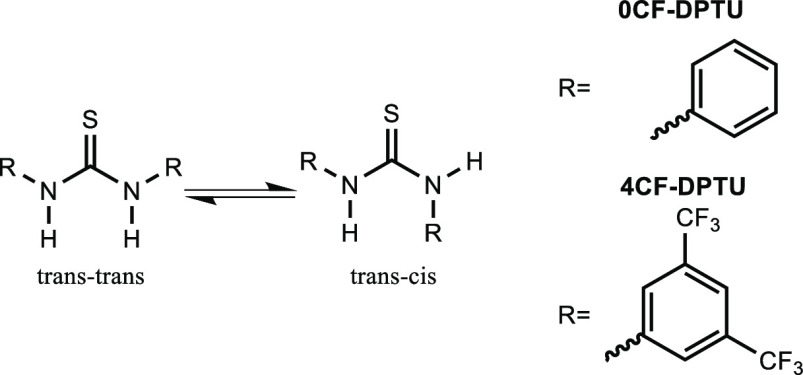
*Trans*–*trans* and *trans*–*cis* conformation of the studied diphenylthiourea (DPTU)-based catalysts.

In principle, each thiourea N–H group can adopt two predominant conformations, with the thioamide hydrogen and the thiocarbonyl in *cis*- or *trans*-configuration. For the rather poor catalyst diphenylthiourea (0CF-DPTU, Figure 1),^10,12^ conformations with at least one N–H group in *cis*-configuration have been suggested to be favored.^20,21^ Alkyl substituents can further stabilize this conformation via dispersion interactions.^18^ Conversely, the catalytically superior^10,12^ 3,5-bis(trifluoromethyl)phenyl-substituted thiourea (4CF-DPTU, Figure 1) has been suggested to prefer the *trans*-conformation for both N–H groups at room temperature.^15^ In this configuration, both N–H groups of thiourea can interact with a single hydrogen-bond acceptor site of the reacting electrophile, which has been proposed to enhance catalytic activation.^18^ As such, substituents can affect both the electronic structure and conformational equilibria of the catalyst, which makes it challenging to disentangle the effects of conformation and electronic effects on the catalytic activity.^18,19,22−24^

Nuclear magnetic resonance (NMR) spectroscopy can disentangle the *cis*- and *trans*-conformations of thioureas in solution, provided the lifetimes of the *cis*- and *trans*-conformers are sufficiently long.^18^ The *cis*- and *trans*-conformers of thiourea interconvert via rotation around the C(*S*)-N axis. However, because *cis*–*trans* interconversion is fast at ambient temperature, NMR spectra for bare urea or thiourea dissolved in dimethylsulfoxide show a single, motionally averaged signal due to both N–H protons.^25^ Only at reduced temperatures, the rotation around the C(*S*)-N axis becomes sufficiently slow so that the NMR signals split into disparate resonances.^18^ From this coalescence, a rotational barrier of ∼11 to 14 kcal/mol has been deduced.^25^ A similar coalescence has been reported for the catalytically relevant trifluoromethyl-substituted diphenyl thiourea (4CF-DPTU) dissolved in tetrahydrofuran with a rotational barrier of ∼9 kcal/mol.^15^ The similarity of the rotational barrier is in line with a quantum chemical study, which has predicted the rotation to be hardly affected by the nature of the substituents.^26^ Despite this activation barrier being thermally accessible at room temperature, the *trans*–*trans* conformation has been suggested to prevail at room temperature for 4CF-DPTU, while at reduced temperatures, the *trans*–*cis* conformation seems to dominate.^15^ These studies suggest that conformational equilibria are rather complex, presumably due to the sensitivity to inter- and intramolecular dispersion interactions.^18,21,22^ This sensitivity to the exact details of inter- and intramolecular interactions is also reflected in the predictions made by density functional theory calculations: Depending on the functional or the continuum solvent used in these calculations, the *cis*–*cis*, *cis*–*trans*, or *trans*–*trans* conformations have been reported to be energetically most favorable.^15,21,22^ As such, the prevailing conformation at catalytically relevant ambient temperatures remains elusive.

**Figure 2:**
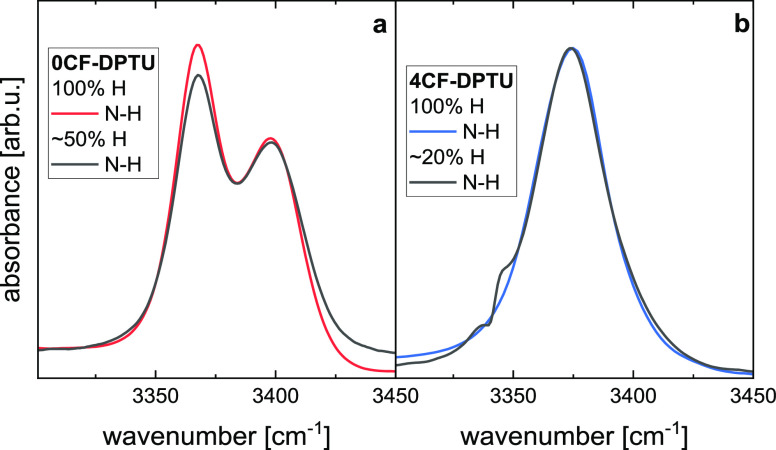
(a) Infrared absorption spectra at N–H stretching frequencies for 0CF-DPTU (red solid line) and isotopically substituted (∼50% H) 0CF-DPTU dissolved in dichloromethane. (b) N–H stretching band for 4CF-DPTU (blue solid line) and isotopically substituted (∼20% H) 4CF-DPTU dissolved in dichloromethane. Spectra were scaled after subtraction of the absorption of the solvent and of a constant background.

To study conformational equilibria on faster timescales, vibrational spectroscopies are a powerful tool, if different conformers have different vibrational signatures. For solutions of methyl- or ethyl-substituted urea^20^—but not thiourea^27^—two disparate N–H stretching bands have been observed, which have been assigned to the *cis*- and *trans*-conformations. Upon substitution with more bulky substituents (e.g., *t*-butyl) also for thiourea two well-separated N–H stretching bands are discernable in the infrared absorption spectra.^27,28^ Similarly, two N–H stretching bands have been observed for the catalyst diphenylthiourea (0CF-DPTU) in carbontetrachloride and dichloromethane.^20,29^ Conversely, the catalytically superior 3,5-bis(trifluoromethyl)phenyl-substituted thiourea (4CF-DPTU) exhibits only a single absorption band at N–H stretching frequencies (see also Figure 2 below).^29^ The formation of hydrogen bonds of the N–H groups—as proposed in the catalytically reactive intermediates—gives rise to markedly red-shifted N–H stretching bands.^29^ Also, other vibrational modes are sensitive to hydrogen-bond formation, and the dichroism of these vibrational modes has the potential to unambiguously determine thioureas’ conformational state^23,24,30^ via comparison to computational spectra, but achieving quantitative agreement is still challenging.^23^ Conversely, achieving agreement between the simulation and experimentally measured vibrational spectra, vibrational spectroscopy has the potential to elucidate conformational equilibria of thiourea catalysts and association equilibria of these catalysts with reactive electrophiles at ambient temperatures.

While the analysis of the hydrogen-bonded N–H band has allowed quantifying the overall binding strengths of the catalysts with the substrates,^29^ the presence of various overlapping infrared absorption bands has prohibited isolating the contributions of the individual molecular species (conformations and reactive intermediates) on the basis of infrared spectroscopy: In the presence of hydrogen-bond acceptors, the hydrogen-bonded N–H stretching band can dominate the vibrational spectra at N–H stretching frequencies and the broad, red-shifted N–H stretching band of hydrogen-bonded thiourea moieties overlaps with the bands of the N–H modes of the nonbonded N–H groups.^29,31,32^ However, the different molecular level conformations and binding states not only alter the infrared resonance frequencies and lineshapes but also result in different interactions with, and thus different vibrational coupling to, the environment. As such, different molecular species can be disentangled using nonlinear infrared spectroscopies even if their spectral contributions are highly congested in the (linear) infrared absorption spectra.^33,34^ Moreover, femtosecond infrared spectroscopy (fs-IR)^33,35−38^ can provide insights into dynamics occurring on timescales of vibrational energy relaxation.

Here, we report a fs-IR study to disentangle different molecular species by probing the N–H stretching bands of diphenylthiourea (0CF-DPTU) and CF_3_-substituted diphenylthiourea (4CF-DPTU) in solution. By performing density functional theory (DFT) calculations and DFT-molecular dynamics (MD) simulations, we show that both, thiourea’s conformation and its interaction with the solvent, affect vibrational bands and our results allow for a precise assignment of the observed bands. Using fs-IR spectroscopy, we show that these different vibrational modes can be disentangled based on the different vibrational relaxation times. We find that for 0CF-DPTU and for 4CF-DPTU, both conformational states (*cis* and *trans*) are populated, and 0CF-DPTU favors the *cis*–*trans* conformation, while 4CF-DPTU favors the *trans*–*trans* conformation. To study hydrogen bonding within reactive intermediates, we use 1,3-diphenyl-2-propenone as a model electrophile of Diels–Alder cycloadditions.^10^ Transient infrared spectra indicate that vibrational relaxation of the N–H stretching mode is significantly accelerated upon hydrogen-bond formation to the ketone, which allows isolating the spectral signatures of the hydrogen-bonded complexes. Analysis of the different spectral contributions provides evidence for only one N–H group forming a strong hydrogen bond to the ketone. This hydrogen bond exchanges on a timescale of a few picoseconds for 0CF-DPTU. For 4CF-DPTU, this exchange appears to occur much faster. Together, our results suggest that the reduced catalytic activity of 0CF-DPTU relative to 4CF-DPTU originates from different conformational equilibria and bonding lifetimes, rather than from different binding strengths between the catalyst and the substrate.

## Experimental Methods and Simulation Procedure

### Sample Preparation

Diphenylthiourea (**0CF-DPTU**, Fluka, >98%), *N,N′*-bis[3,5-bis(trifluoromethyl)phenyl]-thiourea (**4CF-DPTU**, TCI, >98%), 1,3-diphenyl-2-propenone (Sigma-Aldrich, >98%), and dichloromethane (DCM, Fischer Chemical, 99.98%) were used as received. The solutions were prepared by weight using an analytical balance, assuming the solution density to be the density of the solvent. For isotopic substitution experiments, DPTUs were dissolved in methanol-d4 and the solvent was evaporated in vacuo after 12 h. The deuterated DPTUs were then dissolved in DCM, which was stored over a molecular sieve (4 Å) prior to use.

### Linear Infrared Spectroscopy

A Bruker Vertex 70 and a Nicolet 850 Magna IR spectrometer were used to record the infrared absorption spectra with a spectral resolution of 4 cm^–1^. The sample was kept between two CaF_2_ windows, separated by a 0.2 or 0.5 mm thick spacer in a “Specac demountable Omni Cell”.

### Femtosecond IR Spectroscopy

Femtosecond IR pump-probe experiments^39,40^ were based on a Ti:sapphire regenerative amplifier that generates 800 nm pulses at a repetition rate of 1 kHz (Spectra Physics, Spitfire Ace). First, 1.5 mJ of the 800 nm pulses are used to pump an optical parametric amplifier (TOPAS Prime with noncollinear difference-frequency generation, Light Conversion), which generates IR pulses centered at ∼3000 nm, with a pulse duration of ∼100 fs, a pulse energy of 5–8 μJ, and a full width at half maximum (FWHM) of ∼400 cm^–1^. The IR pulses are split into a pump pulse (∼92%), a probe pulse (∼4%), and a reference pulse (∼4%) using a wedged CaF_2_ window. The pump beam is guided to a delay stage to control the time delay between pump and probe pulses. Using a λ/2-plate, we rotate the polarization of the pump beam to 45° with respect to the probe pulse. The pump beam is modulated at 500 Hz using a mechanical chopper wheel. With a parabolic mirror, all three beams are focused into the sample, and pump and probe beams are overlapped at this position. The pump beam is blocked after the sample and the probe and the reference beams are re-collimated and guided through a rotating wire-grid polarizer, which enables one to select polarization components of the probe beam parallel or perpendicular to the pump beam. Probe and reference beams are focused into a spectrograph (Horiba, Triax 180, 300 or 150 L/mm) and spectrally dispersed on two lines of a 2 × 32 pixel nitrogen-cooled MCT detector (InfraRed Associates Inc.). Transient spectra were obtained from the modulated probe intensities and corrected for intensity fluctuations as determined from the reference pulse intensities. Transient spectra were recorded both parallel and perpendicular to the pump pulse polarization and used to construct the isotropic absorption changes, Δα.

### Density Functional Theory Calculations

DFT calculations were performed using Orca^41^ 5.0.3 at the revPBE^42^-D3(0)^47^/def2-TZVPP^43,44^ level of theory applying a polarizable continuum model^45^ (DCM). To explore the conformational degree of freedom, we performed relaxed energy surface scans varying the S–C–N–C dihedral angle of one phenyl-thiourea group from 0 to 180° at increments of 10°. Further geometry optimizations were performed starting from 0 and 180° in the continuum solvent using different functionals (PBE^46^-D3(BJ),^47,48^ M06,^49^ and CAM-B3LYP^50^-D3(BJ)^47,48^) to compare the energies of the two conformers using different functionals. To warrant convergence, we have used an increased density for the numerical integration grid, as geometry convergence sometimes contained large numerical uncertainties.

### Ab Initio Molecular Dynamics Simulation

We performed BOMD simulations using the CP2K code.^51^ We used the revPBE^42^ exchange–correlation functionals together with the empirical van der Waals correction scheme using Grimme’s D3(0)^47^ correction. We have used the mixed Gaussian and plane wave approach. Atomic orbitals were described using the DZVP basis set with a plane wave density cut-off at 320 Ry. Core electrons were described using norm-conserving Goedecker–Teter–Hutter pseudopotentials.^52^ To accelerate the simulations, we used D atoms instead of H atoms throughout and the time step was set to 1 fs. All simulations were performed at 300 K in the NVT ensemble with the thermostat of the canonical sampling through the velocity rescaling method.^53^ The simulation boxes contained 50 deuterated DCM molecules and 1 deuterated 0CF-DPTU molecule in its *cis*–*trans* or *trans*–*trans* conformation. The length of the box size was set to 17.78 Å. After 17 ps equilibration, we obtained 21 ps MD trajectory, where we record the trajectories every 5 fs for computing the vibrational density of states (VDOS) based on the N–H bond velocities.

## Results and Discussion

### N–H Vibrational Structure and Dynamics of Thiourea Catalysts in Solution

#### Experimental N–H Stretching Vibrational Structure

To explore the effect of CF_3_ substitution on the conformational state and the vibrational structure and dynamics of diphenylthiourea catalysts, we study solutions of the catalysts in DCM. In Figure 2, we show the infrared absorption spectra of the catalysts 4CF-DPTU and 0CF-DPTU at N–H stretching frequencies.^29^ The linear absorption spectrum of 0CF-DPTU (Figure 2a) shows a clear double-peak structure with two modes at ∼3370 and ∼3400 cm^–1^, in line with previous reports on 0CF-DPTU in carbontetrachloride.^20,28^ Conversely, the linear infrared absorption spectrum of 4CF-DPTU (Figure 2b) reveals a single, rather symmetric N–H stretching band at ∼3375 cm^–1^. As will become more apparent below, the assignment of these bands is not straightforward and partly contradicting assignments have been reported previously. Thus, we consider the vibrational structure in more detail.

For both thiourea compounds, two N–H groups are present. Therefore, two molecular level N–H modes will contribute to the vibrational response. Observation of two disparate bands in the infrared absorption spectra may have different origins. On the one hand, (i) coupling of the two N–H groups can give rise to the presence of two stretching modes (similar to symmetric and antisymmetric stretching bands). On the other hand, (ii) the conformational state can result in different resonance frequencies depending on the conformation of the N–H group: e.g., if the N–H groups in the *cis*-conformation have characteristic frequencies separated from those in the *trans*-conformation, one can observe the two distinct vibrational bands in the infrared spectra. For a wide range of amides, substituted ureas, and thioureas, the different bands have been ascribed to (ii) different conformational states, with the higher frequency mode assigned to the *trans*-conformation and the lower frequency band to the *cis*-conformer.^20^ In contrast, a computational study has suggested the opposite assignment for diphenylurea with a minor peak splitting due to (i) coupling.^54^

To establish the contribution of coupling to the N–H bands of the herein studied thioureas, we perform isotopic substitution experiments: Upon partial substitution of N–H by N–D, the resonance frequencies of the N–D groups shift to lower frequencies (see discussion of the N–D bands in the Supporting Information, SI, Figure S1), thus markedly reducing intramolecular coupling^55^ between N–H (N–D) groups. Figure 2a compares the spectra for 0CF-DPTU to the spectra of isotopically substituted 0CF-DPTU for which ∼50% of the N–H groups have been substituted by N–D. Figure 2a illustrates that the N–H stretching band for 0CF-DPTU coincides with the N–H stretching band of isotopically substituted 0CF-DPTU. The same is true for the N–H stretching band of 4CF-DPTU (Figure 2b). Based on this similarity, we conclude that coupling between N–H groups does not affect the lineshape of the N–H stretching band of 0CF-DPTU and 4CF-DPTU.

Above, the experimental infrared spectra indicate that coupling between N–H modes is weak, and the observed lineshapes are solely due to different conformational states of the studied thioureas. For 4CF-DPTU, only one N–H band is detected in the IR spectra. The presence of a single N–H mode might be due to the prevalence of a single conformer—consistent with the *trans* conformation dominating for 4CF-DPTU at room temperature^15^—or due to coinciding vibrational bands of different conformers, which finds support by the gradual shift of the N–H bands with increasing CF_3_ substitution.^29^ For 0CF-DPTU, two vibrational modes can be clearly observed, indicative of two distinct conformational states, yet the so far reported assignment to molecular-level groups is, as outlined above, ambiguous.

#### Computational Assignment of the N–H Stretching Bands

**Figure 3:**
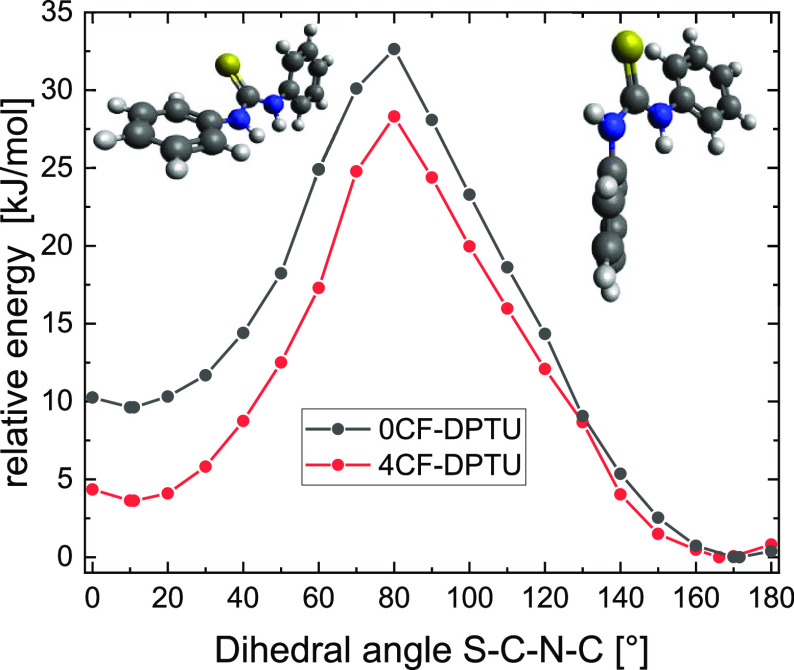
Relative energy of 0CF-DPTU (black symbols) and 4CF-DPTU (red symbols) as a function of the S–C–N–C dihedral angle as obtained using density functional theory calculations (revPBE-D3(0)). Ball-stick models show the obtained conformations for 0CF-DPTU at dihedral angles of 0° (left) and 180° (right).

To explore the peak assignments, we performed DFT calculations based on the dispersion corrected revPBE functional in DCM as a continuum solvent. We first consider the energetic differences between the different conformers: In Figure 3, we show the relative energy as a function of the S–C–N–C dihedral angle (Θ) for 0CF-DPTU and 4CF-DPTU. We find the *cis*–*trans* (Θ = 180°) conformer of 0CF-DPTU to be ∼9.6 kJ/mol more stable than the *trans*–*trans* (Θ = 0°) conformer. For 4CF-DPTU, the energetic difference between both conformers is lower and the energy of the *cis*–*trans* conformation is ∼4.5 kJ/mol lower than that of the *trans*–*trans* conformation. We note that we obtained very similar energetic differences using the PBE-D3(BJ) level of theory (0CF-DPTU: 8.7 kJ/mol; 4CF-DPTU: 2.8 kJ/mol), the CAM-B3LYP-D3(BJ) level of theory (0CF-DPTU, 8.6 kJ/mol; 4CF-DPTU: 2.6 kJ/mol), or the nondispersion corrected M06 functional, as used in ref (15) (0CF-DPTU, 5.2 kJ/mol; 4CF-DPTU: 0.3 kJ/mol). Albeit the absolute conformational energies using DFT calculations depend on the computational method, in line with very detailed earlier studies,^56^ our findings using the revPBE functional broadly agree with earlier reports on 4CF-DPTU.^22^ In general, the calculations consistently show that the *trans*–*trans* conformation becomes more favorable for 4CF-DPTU, as compared to 0CF-DPTU. We also find the activation barrier for rotation around the C–N axis (at Θ ≈ 80°, Figure 3) for 0CF-DPTU and 4CF-DPTU to be very similar, making faster conformational exchange dynamics^57^ for one of the thioureas unlikely. Hence, the DFT calculations indicate that for both thioureas, both conformational states are thermally accessible, yet their energetic difference will lead to different relative populations.

We explore how these different conformations can be distinguished in the vibrational spectra based on their harmonic frequencies at the DFT level of theory. The DFT calculations indeed predict the N–H stretching frequencies to report on these conformations: For 0CF-DPTU in the *trans*–*trans* conformation, the two N–H modes are located at 3480 and 3487 cm^–1^. For 4CF-DPTU in the *trans*–*trans* conformation, these frequencies are very similar (3477 and 3486 cm^–1^). The small difference in the frequency of both modes suggests weak vibrational coupling, consistent with the insensitivity of the experimental bands to isotopic substitution (see Figure 2).

Conversely, for the *cis*–*trans* conformation, we find a larger frequency separation of the two N–H stretching modes for both 0CF-DPTU (*cis* N–H: 3468 cm^–1^; *trans* N–H: 3517 cm^–1^) and 4CF-DPTU (*cis* N–H: 3472 cm^–1^; *trans* N–H: 3507 cm^–1^). Hence, these DFT calculations confirm our conclusions from the experimental spectra that conformational equilibria strongly affect vibrational bands. However, while our DFT calculations suggest that the lower frequency band is due to the N–H group in the *trans* position—in line with earlier computations on substituted ureas^54^—it is at variance with most of the previous experimental studies, which have arrived at the opposite assignment.^20,27,28^

**Figure 4:**
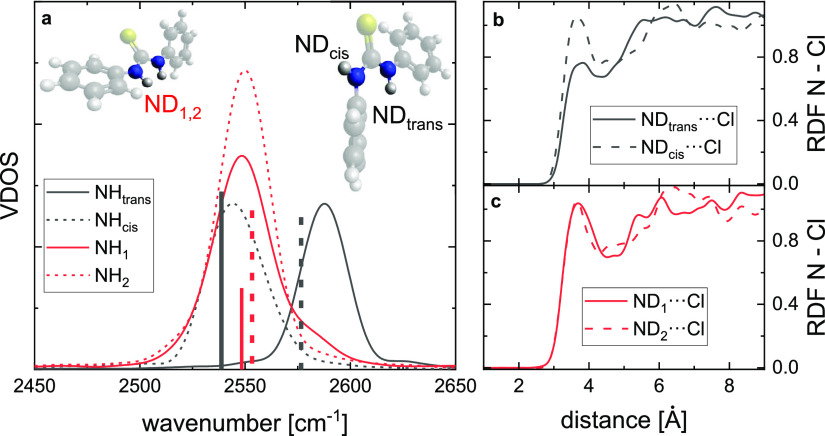
(a) Vibrational density of states (VDOS) for the N–D stretching coordinate of both 0CF-DPTU N–D groups as obtained from ab initio molecular dynamics simulations of the *trans*–*trans* conformer (red lines) and of the *cis*–*trans* conformer (black lines). Vertical lines show the corresponding squared transition dipole moments for the normal modes as obtained from DFT calculations (revPBE-D3(0)) in a continuum solvent. In panels (b) and (c), the interaction of the solvent with the different N–D groups is shown by the N–Cl radial distribution functions for the *cis*–*trans* and the *trans*–*trans* conformer, respectively.

To resolve this conflicting assignment, we assess the effect of the solvent: Besides the electronic structure affecting the N–H bond strengths, also interactions with the solvent affect the N–H bonding potential, i.e., its vibrational frequencies. To account for the solvent explicitly, we perform ab initio MD simulations of deuterated 0CF-DPTU in its *cis*–*trans* and its *trans*–*trans* conformation dissolved in deuterated DCM. We find that the center of the N–D stretching VDOS for the *trans*–*trans* conformer agrees well with the harmonic normal modes of the DFT calculations (Figure 4a). Conversely, the VDOS predicts the N–D group in the *trans* position of the *cis*–*trans* conformer to have a higher stretching frequency, while the frequency of the *cis* N–D group virtually coincides with the VDOS of the *trans*–*trans* conformer. As such, the DFT calculations and the ab initio MD simulations result in the opposite assignment of the higher and lower frequency infrared bands to the *cis* and *trans* N–D group (Figure 4a). These opposing frequencies can be explained by the interaction with the solvent DCM: The first coordination peak in the N–Cl radial distribution function is markedly lower for the *trans* N–D group compared to the *cis* N–D (Figure 4b), evidencing sterically hindered N–D–solvent interaction for the *trans* N–D. The weaker interaction of the *trans* N–D results in the blue-shift of its stretching frequency but only for the *cis*–*trans* conformer. For the *trans*–*trans* conformer, both N–D groups in *trans* conformation are well accessible to the solvent (Figure 4c) and therefore vibrate at lower frequencies.

Together, the ab initio MD simulations lead to a revised band assignment as compared to previous experimental^20,27,28^ or computational^54^ reports: The higher frequency band (Figure 2) is due to N–H (N–D) groups in the *trans* configuration of 0CF-DPTU in the *cis*–*trans* conformation. The lower frequency band is due to the *cis* N–D group of the *cis*–*trans* conformer and due to the N–D groups of the *trans*–*trans* conformer. Assuming similar transition dipole moments for both bands, the experimentally observed slight excess of the amplitude of the lower-frequency band (Figure 2) indicates that the *cis*–*trans* conformer is the prevailing conformation of 0CF-DPTU and the *trans*–*trans* conformer the minor conformer. Though this prevalence of the *cis*–*trans* conformer for 0CF-DPTU contrasts previous conclusions from spectroscopic data,^20,27,28^ it is consistent with this conformation being energetically favored (Figure 3).

#### Vibrational Dynamics of the N–H Stretching Modes

To better resolve different contributing vibrational modes to the observed infrared absorption bands experimentally, we performed fs-IR experiments. In these experiments, the N–H stretching modes are excited with an infrared pump pulse, and the time-dependent changes of the absorption spectrum induced by the pump pulse are detected with a variably delayed probe pulse. The thus obtained isotropic transient absorption spectra allow us to monitor the spectrally resolved vibrational dynamics.^58,59^

**Figure 5:**
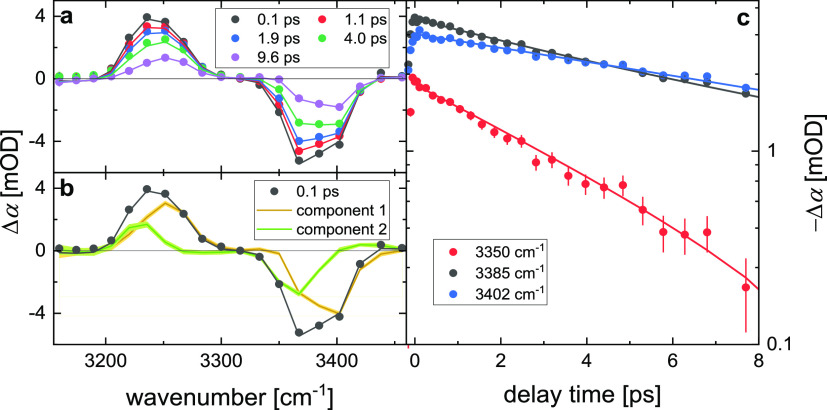
(a) Isotropic transient infrared spectra at N–H stretching frequencies at selected delay times for 20 mM 0CF-DPTU dissolved in dichloromethane. Symbols show experimental data, error bars are smaller than the symbol size, and solid lines show the fit with the kinetic model (see the text). (b) Isotropic transient infrared spectrum at 0.1 ps delay time (symbols) together with the fit (solid black line). The orange and the green lines display the contribution of both spectral components as extracted from the fit. Fit uncertainties are displayed as shaded areas. (c) Transient signals at selected probing frequencies as a function of delay time. Symbols correspond to experimental data, error bars show the shot-to-shot standard deviation, and solid lines show the kinetic model fit.

For solutions of 0CF-DPTU in DCM, the transient spectra at early delay times (0.1 ps in Figure 5a) show a bleaching signal centered at the fundamental frequency of the N–H bands (Figure 2a) due to the ground-state depletion and stimulated emission from the excited state. The corresponding induced excited-state absorption due to the 1 → 2 transition of the excited oscillators is (anharmonically) red-shifted^60^ by ∼130 cm^–1^. We note that with the spectral resolution of the experiments in Figure 5a, the two vibrational modes apparent in Figure 2a are not resolved, yet, give rise to a broad, asymmetric transient signal. Using a higher spectral resolution (see the SI, Figure S2), both modes of 0CF-DPTU can be resolved, yet at the cost of recording the excited-state absorption.

With increasing delay time, the magnitude of the transient signals decays due to relaxation to the vibrational ground state (Figure 5a). Yet, also the spectral shape of the bleaching signal varies with time: the minimum of the bleaching signal is located at ∼3368 cm^–1^ at early delays (<2 ps in Figure 5a) and at ∼3402 cm^–1^ at later delay times (>4 ps in Figure 5a). Such spectral shifts during vibrational relaxation are indicative of frequency-dependent vibrational relaxation times:^59,61^ The transient signals at ∼3368 cm^–1^ decay faster than the bleaching signal at ∼3402 cm^–1^ resulting in an apparent blue-shift of the total bleaching signal with increasing delay time (Figure 5a). These frequency-dependent vibrational dynamics are even more apparent in the delay traces shown in Figure 5c, which demonstrate a markedly faster decay of the bleaching signal for red-shifted N–H groups.

To quantify the frequency-dependent vibrational relaxation, we model the experimental data using a kinetic model based on the excitation of two distinct vibrational states (see the SI).^33,59^ Each state is characterized by its transient vibrational spectrum, and its contribution to the transient spectrum exponentially decays with its characteristic vibrational relaxation time (for details, see Figure S3) to a heated ground state. This model excellently describes the experimental data (see Figure 5), and we find characteristic relaxation times of 11.9 and 3.7 ps for these two states. The spectra associated with both states and their modeled contribution to the transient signals at 0.1 ps are displayed in Figure 5b. These spectra signify two disparate oscillators: a ground-state bleaching signal at ∼3400 cm^–1^ and red-shifted excited-state absorption at 3250 cm^–1^ for the slower relaxing state (component 1 in Figure 5b, decay time of 11.9 ps) and a bleaching signal at ∼3360 cm^–1^ with a red-shifted induced absorption at ∼3230 cm^–1^ for the faster decaying (component 2 in Figure 5b, 3.7 ps decay time) contributions. These spectral shapes again confirm that both modes are not or only very weakly coupled: If they were coupled, excitation of one N–H oscillator would give rise to spectral modulations at the resonance frequency of both modes,^62^ which is not observed in the data in Figure 5. Assuming an equal (50 ± 5%) population of both states at 0 ps delay (see also the SI), we find the magnitudes of both associated spectra to be very similar, which is expected if both N–H modes have similar transition dipole moments. As for 0CF-DPTU, two vibrational modes contribute at N–H stretching frequencies (Figure 2a), and the frequency-dependent vibrational relaxation can be explained by both vibrational modes having disparate relaxation times: the *trans* N–H groups relax markedly slower than the *cis* N–H group (and the N–H groups of the *trans*–*trans* conformer). The slower relaxation of the *trans* N–H group can again be rationalized by weaker interaction with the solvent (Figure 4b) and, thus, weaker coupling to the low frequency modes of the solvent, required for vibrational relaxation.^63^

**Figure 6:**
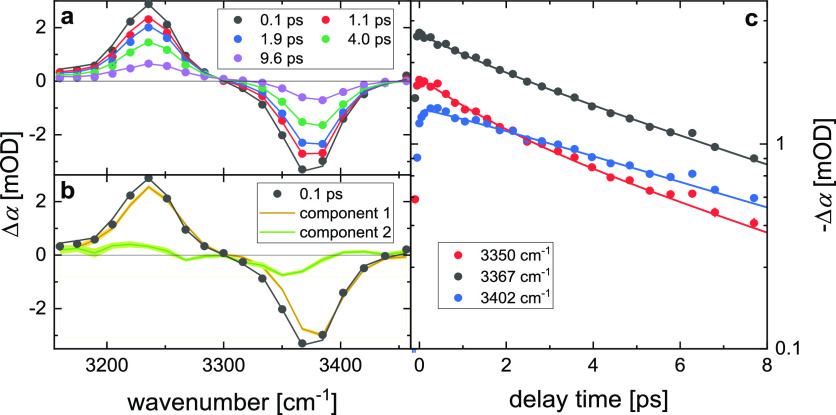
(a) Isotropic transient infrared spectra at N–H stretching frequencies at selected delay times for 20 mM 4CF-DPTU dissolved in dichloromethane. Symbols show experimental data, error bars are smaller than the symbol size, and solid lines show the fit with the kinetic model (see the text). (b) Isotropic transient infrared spectrum at 0.1 ps delay time (symbols) together with the fit (solid black line). The orange and the green line display the contribution of both spectral components as extracted from the fit. Fit uncertainties are displayed as shaded areas. (c) Transient signals at selected probing frequencies as a function of delay time. Symbols correspond to experimental data, error bars show the shot-to-shot standard deviation, and solid lines show the kinetic model fit.

Remarkably, despite the infrared absorption spectrum of 4CF-DPTU displaying only a single N–H band, our infrared pump-probe results for solutions of 4CF-DPTU closely resemble those for 0CF-DPTU. The transient signals for the N–H stretching modes of 4CF-DPTU display a bleaching signal at the fundamental frequency of the N–H mode and an adjacent induced excited-state absorption (Figure 6a). The transient bleaching features of 4CF-DPTU are narrower (∼35 cm^–1^ FWHM at 0.1 ps in Figure 6a) than for 0CF-DPTU (∼45 cm^–1^ FWHM at 0.1 ps in Figure 5a), which reflects the different lineshapes in Figure 2. Despite these narrower linewidths, the time-dependent transient spectra for 4CF-DPTU also display an apparent blue-shift of the bleaching signal with increasing delay time (Figure 5a). Again, this time dependence of the lineshape is indicative of frequency-dependent vibrational energy relaxation. Indeed, also for 4CF-DPTU, the delay traces at three selected detection frequencies in Figure 6c demonstrate that signals at red-shifted frequencies (e.g., at 3350 cm^–1^) decay faster than the transient signals at the blue wing of the N–H band (e.g., at 3402 cm^–1^).

Accordingly, we use the same kinetic model (see the SI) with two vibrationally excited states to fit the data for 4CF-DPTU. Also for 4CF-DPTU, such a model with the two modes relaxing with 6.4 and 1.6 ps describes the data very well (Figure 6). In analogy to our findings for 0CF-DPTU, the transient component spectra display signatures of two uncoupled oscillators. The slower relaxing state (component 1 in Figure 6b, 6.4 ps decay time) displays a bleaching signal at ∼3385 cm^–1^ and an excited-state absorption at 3235 cm^–1^. The faster relaxing state (component 2 in Figure 6b, 1.6 ps decay time) is observed at somewhat red-shifted frequencies (bleach at 3350 cm^–1^, induced absorption at 3220 cm^–1^). However, in contrast to our findings for 0CF-DPTU, the contribution of the red-shifted state is much weaker (as compared to the blue-shifted mode) for 4CF-DPTU: Assuming a 20% population of the red-shifted component and 80% of the blue-shifted state at 0 ps results in nearly equal magnitudes of both component spectra, which would result from equal transition dipole moments of both states (see also discussion in the SI).

Based on the similarity to our findings for 0CF-DPTU, we tentatively assign the red-shifted mode to the *cis* N–H group of the *cis*–*trans* conformer of 4CF DPTU. Its weak contribution to the spectra indicates that the *cis*–*trans* conformer is minor species. Accordingly, the dominating contribution stems from the *trans* N–H groups of the *trans*–*trans* conformer. The fact that both states can be separated based on their vibrational relaxation time shows that spectral diffusion (i.e., the exchange between both N–H modes) occurs on longer timescales than relaxation to the ground state^61^ (>5 ps), which is broadly consistent with slow conformational exchange dynamics.^25,26^ Based on the above estimates of the populations (20 ± 3% *cis* N–H groups, 80 ± 3% *trans* N–H groups), our results indicate that for 4CF-DPTU the *trans*–*trans* conformation prevails (∼60%), yet also the *cis*–*trans* conformer is present in solution (∼40%). These similar populations are also somewhat consistent with their similar relative energies (Figure 3).

Overall, our time-resolved infrared experiments on neat thioureas in solution indicate that the N–H stretching modes of 4CF-DPTU relax faster than those of 0CF-DPTU, indicative of stronger (e.g., anharmonic) coupling to the solvent bath.^64−66^ For both thioureas, we find two vibrational modes with distinctively different energy relaxation times to contribute to the spectra yet with different populations. These oscillators are assigned to the different conformations of the N–H groups of the thioureas, and their relative population is altered in the presence of CF_3_ substituents.

### N–H Hydrogen Bonding in the Presence of Ketones

#### Infrared Absorption Spectra in the Presence of Diphenylpropenone

**Figure 7:**
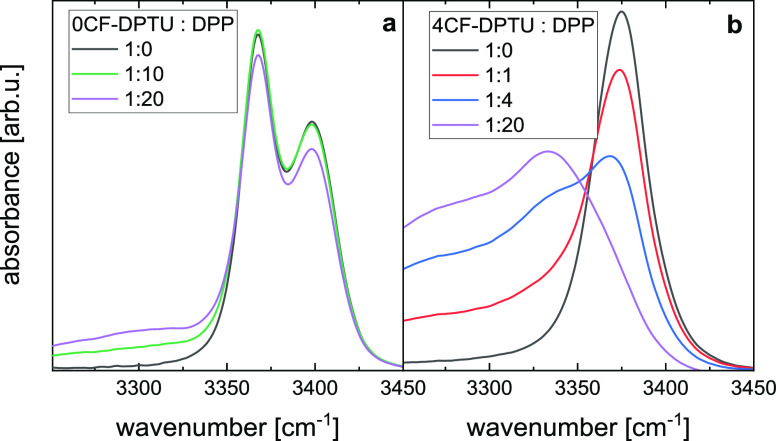
Infrared absorption spectra at N–H stretching frequencies for solutions of 20 mM (a) 0CF-DPTU and (b) 4CF-DPTU in dichloromethane in the presence of diphenylpropenone (DPP) at varying DPTU/DPP molar ratios. All spectra have been corrected for the solvent background and contributions from DPP. Data taken from ref (29).

To investigate the vibrational structure and hydrogen bonding of both thioureas in the presence of electrophiles that are commonly used in catalysis, we study the interaction of 0CF-DPTU and 4CF-DPTU with diphenylpropenone (DPP)^29^ as the representative substrate in solution. For these mixtures, our earlier study^29^ has indicated that the average binding strength of 0CF-DPTU to DPP is ∼10-fold weaker than that of 4CF-DPTU to DPP. This difference in binding strength is also apparent in the infrared absorption spectra shown in Figure 7. For solutions of 0CF-DPTU, a broad absorbance at ∼3300 cm^–1^ emerges with the increasing concentration of DPP (Figure 7a), indicative of N–H stretching modes of N–H groups hydrogen bonded to the C=O group of DPP.^32,67^ Yet, even for a 20-fold excess of DPP, the amplitude of this band is rather low. Conversely, for 4CF-DPTU, already low amounts of DPP give rise to a red-shifted absorbance, and at a 20-fold excess of DPP, the red-shifted spectral feature dominates the N–H stretching band, while the nonbonded band of the catalyst is hardly detectable. Although these spectra have allowed quantifying the average interaction strength of the thioureas with the substrates,^29^ the contributions of individual species are challenging to disentangle due to their overlapping infrared bands.

#### Vibrational Dynamics of 0CF-DPTU–DPP Mixtures

**Figure 8:**
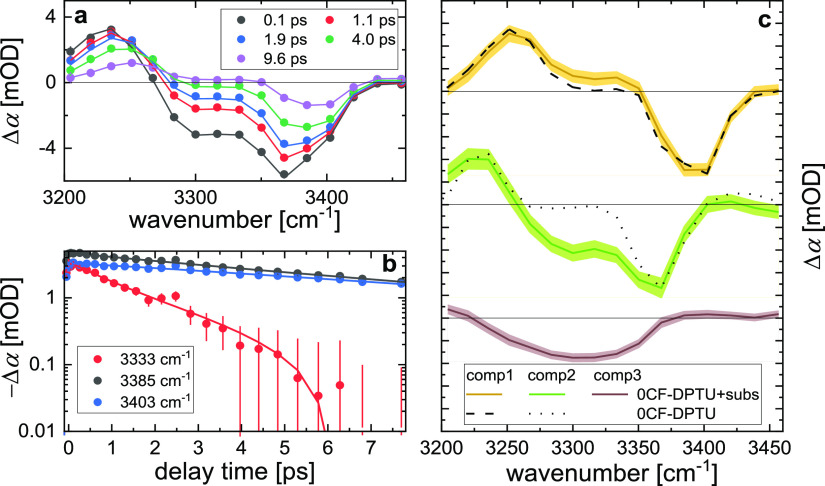
Time-resolved infrared spectroscopy data for a 0CF-DPTU/DPP mixture at a 1:20 molar ratio in dichloromethane. (a) Isotropic transient infrared spectra at N–H stretching frequencies at selected delay times. (b) Transient signals at selected probing frequencies as a function of delay time. Symbols in (a) and (b) correspond to experimental data, error bars show the shot-to-shot standard deviation, and solid lines show the kinetic model fit (for details, see the text). (c) Transient spectra of the three contributing components based on their different vibrational relaxation dynamics. Solid lines show the spectra as extracted from the kinetic model, and shaded areas illustrate the uncertainty of the fit. Solid dashed and solid dotted lines show the two components as observed in the absence of DPP (Figure 5). The three spectra are vertically offset for visual clarity, and solid black lines indicate Δα = 0.

To better discriminate the individual molecular level species contributing to the infrared absorption spectra (Figure 7), we again performed fs-IR experiments. To warrant a sufficiently high contribution of the 0CF-DPTU–DPP complexes, we focus on the 0CF-DPTU/DPP mixture at a 1:20 molar ratio. The transient spectra at different waiting times for this mixture (Figure 8a) resemble those for solutions of the neat catalyst (Figure 5), yet we observe an additional bleaching signal at 3280–3340 cm^–1^. At these frequencies, the absorption band upon addition of DPP emerges (Figure 7a). As such, this additional spectral feature in the fs-IR experiments can be ascribed to the stretching band of N–H groups hydrogen bonded to DPP. Figure 8b demonstrates that the transient signals at these frequencies decay markedly faster than the signals characteristic to the N–H band of free (non-hydrogen-bonded) N–H groups: The transient signals at 3333 cm^–1^ decay ∼4 times faster than the transient signals at 3385 and 3403 cm^–1^ (Figure 8b). As such, the excited population of hydrogen-bonded N–H groups relaxes faster to the vibrational ground state, indicative of stronger anharmonic coupling to lower-frequency modes as commonly observed for hydrogen-bonded systems.^59^

In analogy to the analysis of the data for solutions of neat catalysts, we use a kinetic model to quantify vibrational dynamics and extract the associated spectral contributions (see the SI). To account for the faster relaxing hydrogen-bonded species, we assume three disparate states that relax to a common (heated) ground state. In this manner, we account for the contribution of the hydrogen-bonded species, in addition to the species contributing to the spectra for solutions of neat 0CF-DPTU. To reduce the number of adjustable parameters, we constrain the vibrational relaxation time for two components to those found in solutions in the absence of DPP (11.9 and 3.7 ps). This model excellently describes the experimental data (Figure 8a,b), and we find the third spectral component (the hydrogen-bonded N–H groups, see below) to relax with a characteristic relaxation time of 0.9 ps.

The associated spectra of the slowly relaxing contribution (*trans* N–H group of *cis*–*trans* isomers, see above) virtually coincide with the spectrum extracted from the data on the neat catalyst solutions (component 1, Figure 8c). The fastest relaxing species (0.9 ps relaxation time, component 3, Figure 8c) exhibits a broad bleaching signal at ∼3300 cm^–1^ with the onset of the excited-state absorption visible at 3200 cm^–1^. Hence, as already concluded from the linear absorption spectra, the N–H stretching band of hydrogen-bonded 0CF-DPTU–DPP complexes gives rise to a significantly broadened vibrational mode at lower wavenumbers.

Remarkably, the associated spectrum with the intermediate vibrational relaxation time (3.7 ps) markedly differs in the mixture from the spectrum found in the absence of DPP: In addition to the narrow bleaching signal at ∼3360 cm^–1^ and red-shifted induced absorption at ∼3230 cm^–1^, we find a broad bleach at ∼3250 to 3320 cm^–1^ (Figure 8c, component 2). We note that we obtain the same conclusions without constraining the relaxation times of components 1 & 2. As such, our data suggest that the spectral contributions decaying with 3.7 ps have an additional bleaching contribution in the presence of DPP.

Strikingly, the spectral components of the intermediately relaxing contribution resemble a linear combination of the spectrum found for the neat catalyst (dotted line in Figure 8c) and the hydrogen-bonded species (purple line in Figure 8c). As such, the component spectra assigned to the neat catalyst *trans*-NH groups of the *trans*–*trans* conformer and the *cis* N–H groups of the *cis*–*trans* conformer spectrally mix with the signatures of the N–H groups bound to DPP. Such spectral mixing can be explained by chemical exchange dynamics:^68^ If an initially excited non-hydrogen-bonded N–H group forms a hydrogen bond to the substrate before relaxation to the ground state, the spectral components decaying with this relaxation time will contain significant contributions of the hydrogen-bonded species. We note that also (excitation) energy transfer from the excited N–H group to the neighboring (not excited N–H) group could give rise to similar spectral features.^69^ However, such energy transfer requires coupling between these N–H groups, which is weak for the neat catalyst (see above), and is thus unlikely to be stronger in the presence of DPP, where the resonance frequencies of the two N–H groups are even further apart. As such, the component spectrum in the mixture provides evidence for hydrogen-bond formation dynamics on a < 10 ps timescale. These exchange dynamics are most apparent for the formation dynamics, as the transition dipole moments of the hydrogen-bonded species are high and give rise to a strong signal.^70^ Given the low concentration of 0CF-DPTU (20 mM) and DPP (400 mM) and the large molecular volume of these molecules, their diffusion in solution is slow. Therefore, significant formation of thiourea–DPP complexes from initially nonbonded 0CF-DPTU on a < 10 ps timescale is rendered unlikely. However, a marked asymmetry in the hydrogen-bonding strengths of two N–H groups of 0CF-DPTUs can explain our observations: if only one N–H group of the two nearby N–H groups of 0CF-DPTU in the *trans*–*trans* conformation strongly hydrogen-bonds to DPP, while the other N–H group interacts only weakly with DPP, exchange of the bonding N–H group of 0CF-DPTU can occur on much shorter timescales. In fact, DFT calculations indicate that the hydrogen-bonding strengths of the two N–H groups in 0CF-DPTU–DPP complexes are governed by the balance between π–π and hydrogen-bonding interactions between 0CF-DPTU and DPP and can therefore be very different (see discussion in the SI, Figure S4). Additional support for this scenario is provided from the experiments on 4CF-DPTU (see below). We note that for 0CF-DPTU, this scenario requires the conformational equilibrium of 0CF-DPTU to be shifted toward the *trans*–*trans* conformation when binding to DPP, which appears plausible based on the above findings that interaction with the environment markedly affects conformational states (see Figure 4). In fact, there have been indications for such conformational changes for the structurally very similar bis(α-phenylethyl)thiourea in polar, hydrogen-bond-accepting dimethylsulfoxide.^23^ As such, our data provide evidence for only one N–H group of 0CF-DPTU in its *trans*–*trans* conformation forming a hydrogen bond to DPP and the exchange dynamics of this N–H group occurring on timescales shorter than ∼10 ps.

#### Vibrational Dynamics of 4CF-DPTU–DPP Mixtures

**Figure 9:**
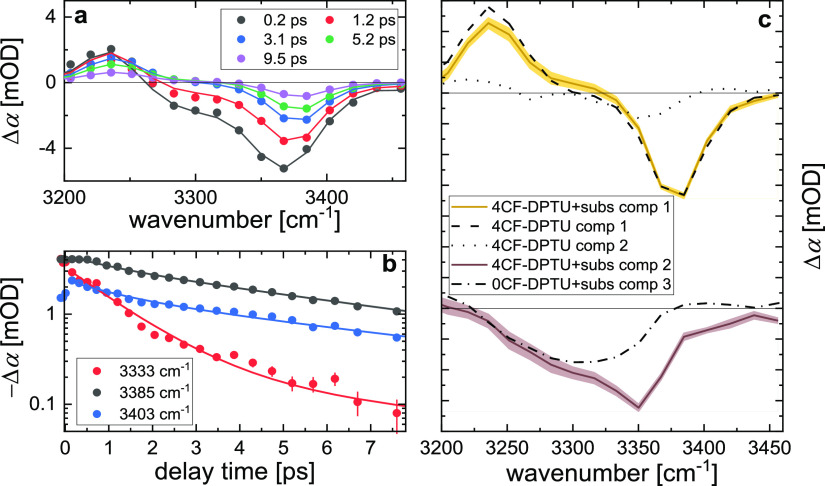
Time-resolved infrared spectroscopy data for equimolar mixtures of 20 mM 4CF-DPTU and DPP in dichloromethane. (a) Isotropic transient infrared spectra at N–H stretching frequencies at selected delay times. (b) Transient signals at selected probing frequencies as a function of delay time. Symbols in (a) and (b) show experimental data, error bars show the shot-to-shot standard deviation, and solid lines show the kinetic model fit (for details, see the text). (c) Transient spectra of the two contributing components, extracted based on their different vibrational relaxation dynamics. Solid lines show the spectra, and shaded areas illustrate the uncertainty of the fit. Solid dashed and solid dotted lines show both components as obtained from the data in the absence of DPP (Figure 6). The dashed–dotted solid line displays the hydrogen-bonded component in 0CF-DPTU/DPP mixtures. Spectra are vertically offset for visual clarity, and solid black lines indicate Δα = 0.

We proceed with studying the vibrational dynamics of 4CF-DPTU–DPP mixtures. To allow for a better comparison to the data for 0CF-DPTU, we focus here on a solution of an equimolar 4CF-DPTU–DPP mixture, for which the absorbances at red-shifted frequencies due to hydrogen-bonded N–H groups are comparable in magnitude (Figure 7). In Figure 9, we show the transient infrared data for this mixture. Similar to the data in Figure 8, we find a broad bleaching signal at ∼3300 cm^–1^ in addition to the ground-state bleaching signal at ∼3380 cm^–1^ and the excited-state absorption at 3240 cm^–1^ (Figure 9a). This broad bleaching signal also decays faster than the transient signal that is also present in the absence of DPP (Figure 9b), in line with our findings for 0CF-DPTU.

Using the same kinetic model based on three excited states, as used to describe the data for 0CF-DPTU, the modeled data for 4CF-DPTU–DPP mixtures converged to two states having the same relaxation dynamics. As such, three different vibrationally excited states—as found for the 0CF-DPTU/DPP mixtures—cannot be discriminated based on their different vibrational lifetimes for 4CF-DPTU/DPP mixtures. We note that this most likely means that the faster decaying species found for solutions of neat 4CF-DPTU (Figure 6) cannot be disentangled due to its weak contribution to the transient signals or due to a shift of the conformational equilibrium toward the *trans*–*trans* conformation in the presence of DPP. Accordingly, we model the data for the 4CF-DPTU–DPP mixture using two excited states decaying to a common vibrational ground state. From this model (see fits in Figure 9a,b), we find that the data can be excellently described assuming two spectral components decaying with 6.4 and 1.2 ps. The associated spectrum of the slowly decaying component coincides with the spectra found for the catalyst in the absence of DPP (cf. component 1 to the dashed and dotted lines in Figure 9c). Hence, the vibrational structure and dynamics in the mixture equal those of the neat catalyst solution, consistent with this component arising from the fraction of 4CF-DPTU not interacting with DPP.

The bleaching signal of the faster-decaying component for 4CF-DPTU exhibits a red-shift similar to that found for 0CF-DPTU. As such, the hydrogen-bond strength, as judged from the N–H stretching frequency, is similar for 0CF-DPTU and 4CF-DPTU. In contrast to our findings for 0CF-DPTU (dashed–dotted line in Figure 9c), the lineshape of the faster-decaying components is clearly asymmetric for 4CF-DPTU with a minimum at 3350 cm^–1^ and an adjacent broad signal extending to a lower frequency (purple line in Figure 9c, the same spectral signatures are found at higher DPP concentrations, see Figure S5). This asymmetric shape is also apparent in the linear infrared spectra for an (1:20) excess of DPP, where 4CF-DPTU–DPP complexes prevail (Figure 7b).^29^ This asymmetry can—in analogy to our observations for 0CF-DPTU–DPP mixtures—be explained by only one of N–H groups of 4CF-DPTU forming a strong hydrogen bond to DPP, while the adjacent nonbonded N–H group gives rise to the narrow bleaching contribution at 3350 cm^–1^. A combination of the two N–H groups contributing to the observed spectral signatures (e.g., a linear combination of components 2&3 of 0CF-DPTU, Figure 8c) can thus explain the asymmetric lineshape of 4CF-DPTU–DPP complexes. The fact that all spectral signatures associated with these 4CF-DPTU–DPP complexes decay with a single vibrational relaxation time of 1.2 ps could mean that (i) both N–H groups happen to have the same vibrational relaxation time or that (ii) exchange of the hydrogen-bonding N–H group with the C=O group of DPP occurs on timescales faster than the vibrational relaxation time. Given that in all fs-IR experiments reported here, the vibrational relaxation time is very sensitive to the bonding or conformational state, (i) is rendered unlikely. Rather, a fast exchange of the N–H group forming a hydrogen bond to the C=O group of DPP seems more likely and will result in a decay of the spectral signatures of both N–H groups with a common decay time. This faster exchange may be rationalized by the CF_3_ substituents weakening π–π interactions between 4CF-DPTU and DPP and thereby reducing the geometric constraints on the hydrogen-bonding geometry. Therefore, our findings indicate that also for 4CF-DPTU–DPP, exchange of the hydrogen-bonding N–H groups takes place on picosecond timescales, yet faster than for 0CF-DPTU–DPP complexes.

## Conclusions

We use the N–H stretching vibration to elucidate both the conformational state and the binding motif of two different thioureas, moderately catalytically active 0CF-DPTU and highly catalytically active 4CF-DPTU, in solution. Using isotopic exchange experiments and DFT calculations, we show that the conformational state in solution is decisive for the lineshapes of the N–H stretching bands. With the help of ab initio MD simulations, we show that *trans* N–H groups of *cis*–*trans* isomers of 0CF-DPTU interact weaker with the solvent, leading to a blue-shift of the N–H stretching band, while the *cis*-NH group and the *trans*-NH groups of the *trans*–*trans* conformation are accessible to the solvent and therefore resonate at red-shifted frequencies. These different interactions with the solvent bath are corroborated by the vibrational relaxation times: the *trans* N–H groups of the *cis*–*trans* conformer are weakly coupled to the solvent and therefore exhibit much slower vibrational energy relaxation dynamics (11.9 ps), as compared to the red-shifted N–H modes (3.7 ps).

For the catalytically active 4CF-DPTU, it is challenging to disentangle different conformational states based on the absorption spectra at N–H stretching frequencies. Yet, we demonstrate that these can be readily disentangled based on their different vibrational lifetimes in femtosecond infrared spectroscopy experiments. The two conformational states of 4CF-DPTU relax twice faster (6.4 and 1.6 ps) than those of 0CF-DPTU, indicative of stronger coupling to the environment. Analysis of the spectral amplitudes indicates a slight excess of the *trans*–*trans* conformation for 4CF-DPTU in solution.

The addition of the prototypical hydrogen-bond accepting substrate DPP leads to a marked red-shift of the N–H stretching band. Discrimination of the spectral contributions of the hydrogen-bonded intermediates to the mixture spectra based on their different vibrational lifetimes shows that the bonding strength—as judged from the degree of red-shift of the thiourea–DPP complexes—is comparable for both thioureas. The spectra provide for both thiourea/DPP mixtures evidence for only one N–H group of the thiourea catalyst in its *trans*–*trans* conformation, forming a strong (spectrally red-shifted N–H) hydrogen bond to DPP. The bonded and nonbonded N–H groups, however, rapidly exchange: for 0CF-DPTU, our results provide evidence for hydrogen-bond formation of initially nonbonded N–H groups on timescales shorter than vibrational relaxation; for 4CF-DPTU, the asymmetric lineshape of the N–H groups associated with 4CF-DPTU–DPP complexes indicates even faster exchange of the bonding N–H moiety. As such, and in contrast to commonly implied symmetric bonding as the predominant mode of activation, our results imply a marked asymmetry in thiourea–ketone bonding, with the exchange critically differing for the weak and the efficient catalyst. Overall, our results point toward conformational dynamics and hydrogen-bonding dynamics being the origin of the markedly different catalytic activities of both DPTUs, rather than solely the bonding strength.

## Supporting Information Available

ND stretching infrared spectra, femtosecond IR spectra with a higher spectral resolution and different 4CF-DPTU/DPP ratios, details on the kinetic modeling and error estimation, and additional DFT calculations on the hydrogen-bond asymmetry (PDF)
