# Supplementary material for: Elucidating Conformation and Hydrogen-Bonding Motifs of Reactive Thiourea Intermediates
Source: ACS Catal. 2022 Oct 5;12(20):12689–700. doi: 10.1021/acscatal.2c03382 (PMC9594049; doi:10.1021/acscatal.2c03382)
Supplement: Supplementary file 1 — cs2c03382_si_001.pdf [file cs2c03382_si_001.pdf]

Supporting Information

for

Elucidating conformation and hydrogen-bonding motifs of reactive thiourea intermediates

*Amelie A. Ehrhard, Lucas Gunkel, Sebastian Jäger, Arne C. Sell, Yuki Nagata, Johannes Hunger**

Max-Planck Institute for Polymer Research, Ackermannweg 10, 55128 Mainz, Germany

*email: hunger@mpip-mainz.mpg.de

N-D stretching bands of DPTUs in dichloromethane

In the main text we show the N-H stretching bands for 0CF-DPTU and 4CF-DPTU together with the spectra for isotopically substituted DPTUs. The corresponding N-D stretching bands for the isotopically substituted 4CF-DPTU (shown with a scaled frequency axis in Figure S1a), is substantially more structured, with a double peak centered at ~2500 cm^{-1} (scaled ~3430 cm^{-1}) and a red-shifted weaker band at ~2480 cm^{-1} (scaled ~3400 cm^{-1}). For 0CF-DPTU two N-D bands at ~2475 cm^{-1} (scaled ~3390 cm^{-1}) and ~2530 cm^{-1} (scaled ~3460 cm^{-1}) are present, yet with an opposite intensity ratio as compared to the corresponding N-H bands (Figure S1b). This different vibrational structure at N-D frequencies may indicate that coupling/conformational equilibria can be better disentangled for the N-D stretching modes due to different linewidths and the different intensities may point towards nuclear quantum effects. Yet, also Fermi resonances could give rise to a different vibrational structure (e.g. the shoulder in Figure S1a) and the exact intensity ratios of the two bands in Figures S1 & 2a somewhat depend on the subtraction of the solvent background. As such, we refrain from detailed analysis of the N-D stretching bands.

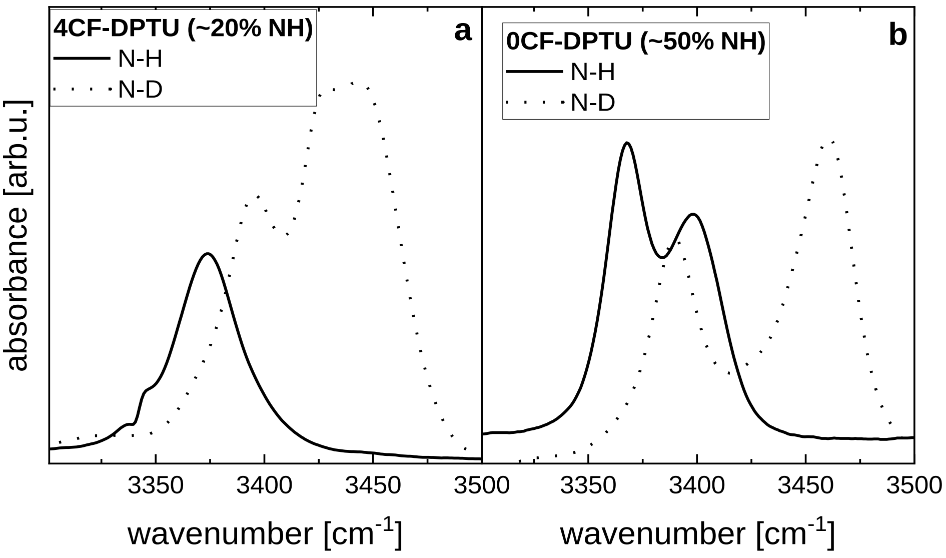

***Figure S1**: N-H stretching bands of isotopically substituted **(a)** 4CF-DPTU (~20% N-H) and **(b)** 0CF-DPTU (~50% N-H) (black solid lines). The black dotted lines show the N-D stretching bands, for which the frequency axis was scaled by a reduced mass factor of* $\sqrt{\frac{2}{16}\frac{15}{1}}$*. From all spectra the absorption of the solvent and a constant background have been subtracted.*

Spectral resolution of the transient signals of 0CF-DPTU

All fs-IR spectra shown in the main manuscript were recorded with a ~15 cm^{-1} spectral resolution to cover all transient spectral signatures (excited state absorption and ground state bleaching signal of all N-H modes), which however does not allow for spectrally resolving the two bands of 0CF-DPTU (see Figure 2, main manuscript). In Figure S2 we demonstrate that the two bands can be resolved using a higher spectral resolution, yet at the cost of detecting the other spectral features. The faster decay of the red-sifted band as compared to the blue-shifted band is also apparent from these data.

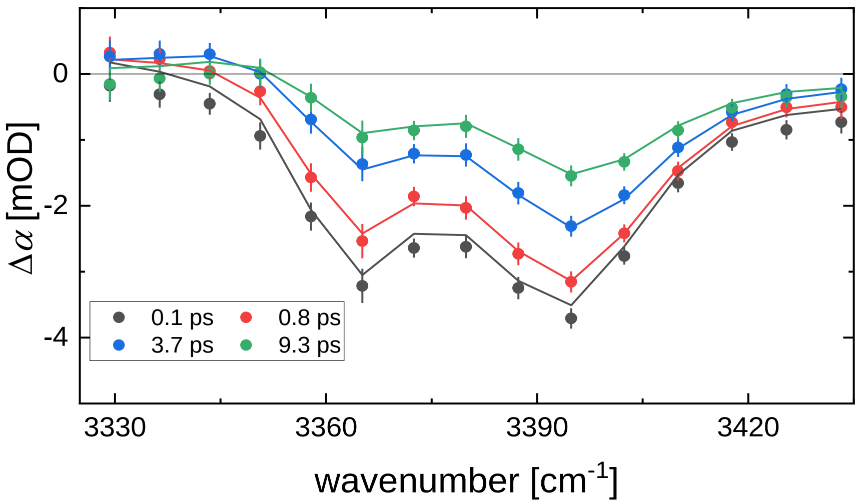

Figure S2: *Isotropic transient infrared absorption spectra at N-H stretching frequencies at selected delay times for 20 mM 0CF-DPTU and 20mM DPP dissolved in dichloromethane. Symbols show experimental data, error bars show the shot-to-shot standard deviation, and solid lines show the fit with the kinetic model.*

Kinetic modelling

To fit the transient absorption data $\Delta\alpha(\omega, t)$, we use kinetic models based on two or three independently decaying populations of excited states (Figure S3), which decay to a common, heated ground state.[1,2] Each state is characterized by its time-independent transient spectrum $\sigma_i(\omega)$, and the time-dependent populations of the excited states are assumed to decay with first order kinetics:

$$N_i(t) = N_i{}^0\, e^{-t/\tau_i} \qquad i = 1 \dots 2 \;\; or \;\; 1 \dots 3 \tag{S1}$$

The initial populations $N_i{}^0$ correspond to the relative population of the excited states right after excitation. The heated ground state, which models weak transient signals (spectral shifts) due to dissipation of the vibrational excess energy, is populated as the vibrational relaxation progresses:

$$N_{\mathrm{heat}}(t) = N_{k+1}(t) = \sum_{i=1}^{k} N_i{}^0 \left(1 - e^{-t/\tau_i}\right) \qquad k = 2 \text{ or } 3 \qquad \text{(S2)}$$

The measured isotropic spectra are modelled by a linear combination of the time dependent population of the three/four states multiplied by their corresponding spectra $\sigma_i(\omega)$:

$$\Delta\alpha(\omega, t) = \sum_{i=1}^{3\ or\ 4} N_i(t)\sigma_i(\omega) \qquad \text{(S3)}$$

The thus extracted spectra of the different contributing spectral components are displayed in Figures 5b, 6b, 8c, and 9c of the main manuscript and the corresponding relaxation times, τ_i, are discussed in the main text.

We note that if the transition dipole moments of all molecular-level excited states are the same, the magnitude of $\sigma_i(\omega)$ associated excited states must be comparable. As such, we adjust the initial excited state populations $N_i{}^0$ such that the maximum bleaching signals of the corresponding $\sigma_i(\omega)$ are the same. Therefore, the relative populations, as discussed in the main text, can be related to the relative abundance of the molecular-level species (N-H conformation). We note that the assumption of similar transition dipole moments, μ, likely holds for the (non-hydrogen-bonded) cis and trans N-H groups of 4CF-DPTU and 0CF-DPTU: For water, the empirically determined transition dipole moment of the OH stretching vibration varies by < 5 % in the frequency range 3370-3400 cm^{-1}.[3] Assuming the same variation for the N-H stretching mode, we estimate the relative uncertainty in the ratio of the μ^4 values of the cis and trans N-H groups to < 20 % (due the $\sigma_i(\omega) \sim \mu_i(\omega)^4$ sensitivity of the fs-IR experiment). Using error propagation, we thus estimate the uncertainty in the populations determined from the fs-IR experiment, which are also given in the main manuscript: The population of the trans N-H groups of the cis-trans conformer of 0CF-DPTU is estimated to 50 ± 5 %. For 4CF-DPTU the population of the trans conformer is accordingly estimated to 80 ± 3 %. The transition dipole moments can, however, be markedly enhanced upon hydrogen-bonding.[3] We therefore refrain from discussing these populations for experiments in the presence of DPP.

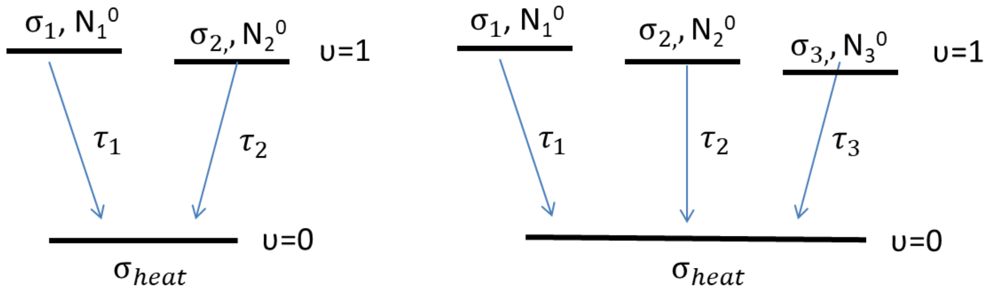

***Figure S3:** Schematic representation of the kinetic models used to describe the experimental isotropic data $\Delta\alpha(\omega, t)$: two component model (left) and three component model (right). Each state is characterized by its transient spectrum σ_i and its initial population $N_i{}^0$. The heated ground state is not populated at $t = 0$ (N_{heat} $(t = 0) = 0$).*

Hydrogen-bonding in 0CF-DPTU – DPP complexes from DFT calculations

In the main manuscript, our fs-IR data provide evidence for largely differing hydrogen-bonding strengths of the two N-H groups of 0CF-DPTU in 0CF-DPTU – DPP complexes. This asymmetry in the hydrogen-bonding strengths is supported by density functional theory calculations of a 0CF-DPTU – DPP complex using the dispersion corrected revPBE functional in dichloromethane as a continuum solvent (see methods section of the main manuscript). Geometry optimization starting from 0CF-DPTU in trans-trans conformation, hydrogen-bonded to the C=O group of DPP results in two very dissimilar N-H ⋯ O distances (d_1 = 2.10 Å and d_2 = 2.59 Å, Figure S4a). The relative energy as a function of d_2, which we determined from a relaxed energy scan upon constraining d_2 at 2.70 Å to 1.80 Å at increments of 0.10 Å starting from the optimized geometry, and from an optimized geometry with short d_2 and gradually shortening d_1, indeed indicates that formation of a hydrogen-bond to only one N-H group is energetically favorable (Figure S4b). This asymmetry of both N-H hydrogen-bonds in the 0CF-DPTU:DPP complex can be explained by π-π interactions of the phenyl rings of DPP and 0CF-DPTU (Figure S4a). In fact, optimizing the geometry of a 0CF-DPTU – acetone complex, where π-π interactions are absent, results in rather similar O-H hydrogen-bonding distances (d_1 = 2.04 Å and d_2 = 2.18 Å, Figure S4c).

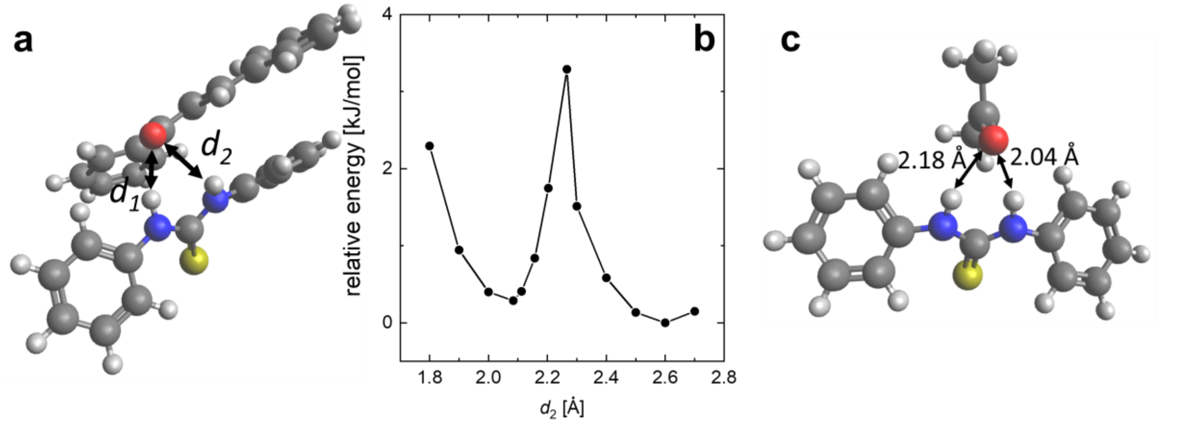

Figure S4:* (a)** *Ball and stick representation of the optimized geometry of a 0CF-DPTU – DPP complex using density functional theory (revPBE-D3(0)). The hydrogen-bond O-H distances are indicated with arrows.* ***(b) *Relative energy of the 0CF-DPTU – DPP complex upon variation of the hydrogen-bond distance* d_2*.* ***(c)*** *Optimized geometry of a 0CF-DPTU – acetone complex. Hydrogen-bonding O-H distances are indicated with arrows.*

Femtosecond infrared data for 4CF-DPTU:DPP at a 1:20 molar ratio

For better comparability of the two thioureas, we use mixtures with similar infrared absorbance in the main text. For 4CF-DPTU equimolar mixtures are presented in Figure 9 of the main manuscript. To better illustrate the asymmetry of the transient infrared spectra associated to the 4CF-DPTU – DPP complexes, we show in Figures S5 transient data for a mixture with a molar excess of DPP (1:20), for which the signatures of the 4CF-DPTU – DPP complexes prevail (Figure 7 in main text, see also Ref. [4]). As can be seen from the data in Figure S5, which were recorded with a higher spectral resolution, the transient spectra display the asymmetric line shape at all times (Figure S5a). The rapidly decaying component (due to the 4CF-DPTU – DPP complexes) dominates the decay of the signal, only at blue-shifted frequencies (3373 cm^{-1} in Figure S5c), where the contribution of the free thiourea is observed (Figure 6 of the main manuscript), the signals decay more slowly at $t > 4$ ps. The same two state kinetic model as used for the equimolar mixture (Figure 9) describes the data very well, and the extracted spectra of the rapidly decaying component (0.8 ps decay time, orange line in Figure S5b) and of the slowly decaying component (6.4 ps decay time, green line in Figure S5b) excellently agree with the spectra found for the equimolar mixture (Figure 9, main manuscript).

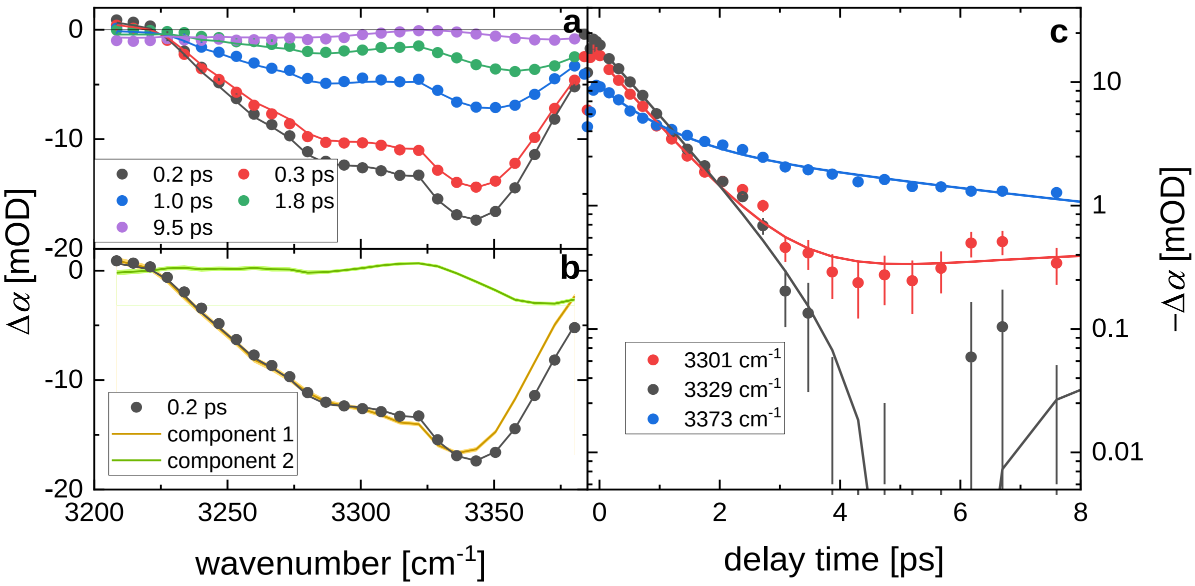

Figure S5: *Time-resolved infrared spectroscopy data for a 4CF-DPTU : DPP mixture at a 1 : 20 molar ratio in dichloromethane.* ***(a)*** *Isotropic transient infrared absorption spectra at N-H stretching frequencies at selected delay times. Symbols show experimental data, error bars are smaller than the symbol size, and solid lines show the fit with the kinetic model (see text)* ***(b)*** *Isotropic transient infrared absorption spectrum at 0.2 ps delay time (symbols) together with the fit (solid black line). The orange and the green line display the contribution of both spectral components as extracted from the fit. Fit uncertainties are displayed as shaded areas.* ***(c)*** *Transient signals at selected probing frequencies as a function of delay time. Symbols correspond to experimental data, error bars show the shot-to-shot standard deviation, and solid lines show the kinetic model fit.*

Supporting References

(1) Lotze, S.; Groot, C. C. M.; Vennehaug, C.; Bakker, H. J. Femtosecond Mid-Infrared Study of the Dynamics of Water Molecules in Water–Acetone and Water–Dimethyl Sulfoxide Mixtures. *J. Phys. Chem. B* **2015**, *119* (16), 5228–5239. https://doi.org/10.1021/jp512703w.
(2) Hunger, J.; Tielrooij, K. J.; Buchner, R.; Bonn, M.; Bakker, H. J. Complex Formation in Aqueous Trimethylamine-N-Oxide (TMAO) Solutions. *J. Phys. Chem. B* **2012**, *116*, 4783–4795.
(3) Loparo, J. J.; Roberts, S. T.; Nicodemus, R. A.; Tokmakoff, A. Erratum to “Variation of the Transition Dipole across the OH Stretching Band of Water” [Chem. Phys. 341 (2007) 218–229]. *Chem. Phys.* **2009**, *361* (3), 185. https://doi.org/10.1016/j.chemphys.2009.06.001.
(4) Ehrhard, A. A.; Jäger, S.; Malm, C.; Basaran, S.; Hunger, J. CF3-Groups Critically Enhance the Binding of Thiourea Catalysts to Ketones – a NMR and FT-IR Study. *J. Mol. Liq.* **2019**, *296*, 111829. https://doi.org/10.1016/j.molliq.2019.111829.
